# Editorial: Microcirculation Guided/Targeted Resuscitation

**DOI:** 10.3389/fmed.2021.649828

**Published:** 2021-03-08

**Authors:** Mihály Boros, Inge Bauer

**Affiliations:** ^1^Faculty of Medicine, University of Szeged, Szeged, Hungary; ^2^Department of Anaesthesiology, University Hospital of Düsseldorf, Düsseldorf, Germany

**Keywords:** shock, microcirculation, mitochondria, resuscitation strategy, oxygen dynamics, hemodynamic coherence

In 1740, Henri-François Le Dran, a French surgeon, observed and described a peculiar state of “*saissisement*,” which commonly follows gunshot wounds. When his treatise was published in London in 1743, this French term was translated as “shock and agitation,” the first noted description of a syndrome “*which suspends the laws of economy for a few moments”* (1). The 280 years since then have seen numerous advances in the mechanism and treatment of this condition through meticulous experimental and clinical research, and our current knowledge on shock categories is deeply rooted in the classical work of George Crile, William Bayliss, Walter Cannon, and Alfred Blalock, to name just a few of the exceptional scientists in the field. Today, their well-established tradition defines shock as circulatory failure, which results in inadequate cellular oxygen utilization and shock conditions, which are characterized by generalized hypoxia/dysoxia or by circulatory dysfunction leading to systemic hypoxia. Nevertheless, shock classifications once thought appropriate and shock mechanisms based on clinical etiologies have been revised numerous times, and today it is perhaps timely to point in new directions again, which would conceivably improve our understanding of this still deadly syndrome.

Of these directions, the importance of microcirculatory investigations should be highlighted first. Like the long evolutionary history of shock research itself, attempts to measure and characterize the performance of human microcirculation have spanned a number of decades, from the first reports on the use of capillary microscopy in 1916 (2) to the first handheld video microscope that provided real-time, dynamic observations at the bedside (3). With these incremental technical advances, it is now recognized that shock-induced peripheral microcirculatory responses are not always connected to macrocirculatory changes, and, likewise, macrohaemodynamic variables cannot always be relied upon to monitor the outcome of shock conditions. Therefore, according to our current understanding, the circulatory consequences of shock can be broadly categorized into those where the macrohaemodynamics are deranged and those where the microcirculation is malfunctioning.

Another related area to be explored in more detail is mitochondrial energetics. The human body employs a wide range of defense mechanisms which react to all types and levels of severity of external or internal insults that disrupt the physiological homeostasis, and, after various durations of compensation, the common denominator of shock states is the failure of energy production essential to maintaining a low-entropy intracellular state. Several reliable techniques are now available to measure the respiratory properties of mitochondria in cells, tissue fractions or whole tissue samples. Protocols for high-resolution respirometry offer sensitive analyses of oxidative phosphorylation and diagnostics for the pathophysiology of a wide array of mitochondrial dysfunctions and with a novel technique (introduced by Baumbach et al. in this issue) non-invasive *in vivo* mitoPO_2_ measurement is also possible.

Substrates and carrier capacity are necessary for the mitochondrion to produce energy. According to this logic, the origins of irreversible, shock-related functional/structural defects should be located at the business end of the cardiovascular system, at the arteriolo-capillary and/or intracellular-mitochondrial junctions. Not surprisingly, the mechanisms of microcirculatory-mitochondrial failures are in the focus of renewed scientific interest. According to this unifying reasoning, whatever the etiology, microcirculatory failure is involved in the syndrome which we call “shock,” and, whatever the mechanism, the mitochondria do not receive sufficient fuel to maintain the energy production necessary for a non-equilibrated cellular metabolic system. This approach may seem to be simplified, but at this stage we can introduce a newly coined term to describe the main components of shock-induced generalized derangements within the circulatory system. According to thought-provoking suggestions by Can Ince, the haemodynamic coherence between the macro- and microcirculation can be defined as “the condition in which resuscitation procedures aimed at the correction of systemic haemodynamic variables are effective in correcting regional and microcirculatory perfusion and oxygen delivery to the parenchymal cells such that the cells are able to perform their functional activities in support of organ function” (4). Most importantly, this interpretation can be expanded to cover the totality of shock pathophysiology, toward the central, macro-, micro-, and subcellular pieces of the patchwork. Figure 1 shows a conceptual framework with connection points where the coherence between the main performers can be lost—and can also be rebuilt, starting from the central pumping station down to the subcellular oxygen dynamics and vice-versa. This might be true in the given coordinates, and, if we accept this holistic view, the final task of a researcher could be to determine the weight of each component in a given timeframe and in a given shock scenario so as to influence the completeness of the “uncoupled” process.

**Figure 1:**
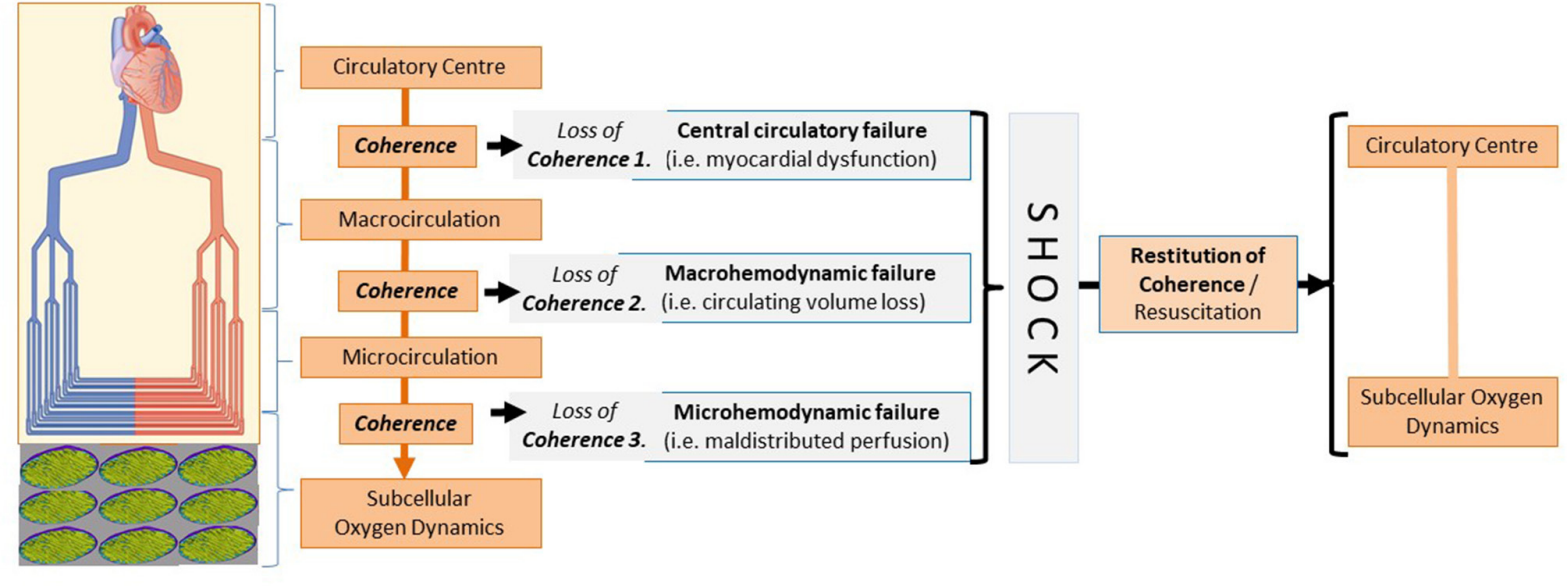
Loss of coherence between the components of hemodynamics - leading to circulatory shock.

We felt especially privileged to write this editorial for a Research Topic that offered high-caliber articles, highlighting many advances in the field. Of the 11 publications, three can be considered to address mechanistic pathways, two to refine existing diagnostic methodologies, and six to improve our understanding of treatment options. In the next part, a brief background outlines the rationale for and novel findings of each study.

## Mechanism

The first article in this series is a thought-provoking paper by Merz et al., where the authors provide a very thorough discussion of a philosophical question (i.e., what to choose for resuscitation in shock conditions, the microcirculation or the mitochondria?) In this line, the review synthesizes our current knowledge of the technical background on how to measure microcirculation and mitochondrial function and the possibilities of microcirculation- or mitochondrion-directed therapies and the advantages and disadvantages of tailoring resuscitation protocols to one or another direction, while the authors remind us that none of the promising microvasculature- or mitochondrial-targeted pre-clinical therapeutic approaches have yet found their way into clinical practice. Readers will find this article of great interest as it presents an excellent review of a critical question and provides many clues, hints, and guidelines for the design of future therapies.

The next two articles are basic science papers that evaluate and further elucidate important mechanistic pathways. Luís et al. provide new insights into the involvement of circulating microRNAs (miRNAs) in endoplasmic reticulum (ER) stress and unfolded protein response (UPR). The authors identified 40 differently upregulated miRNAs in a rodent model of trauma-haemorrhagic shock, of which the vast majority was in close correlation with liver injury markers. Since extracellular vesicles carrying miRNAs have been identified as regulators of intercellular communication, data from these elegant studies indicate that miRNA profiles can provide a rationale for the development of novel therapeutic strategies.

A paper by Warenits et al. reports on the results of a long-term observation study of the pathomechanism of cerebral neurodegeneration caused by cardiac arrest and resuscitation. The authors analyzed several markers of gliosis and parameters of activation of inflammatory and cell-death pathways in the hippocampus and motor cortex in a clinically relevant animal model. Their data revealed decreased haem oxygenase (HO) activity in these brain regions 2 weeks after global ischemia, suggesting causality for HO or HO products in the delayed neurodegenerative processes in these vulnerable brain regions.

### Diagnostics

Methane in the human body can originate in the gastrointestinal tract, and the reduction of mesenteric perfusion is among the first homeostatic responses during internal bleeding. This is the background for a basic science article by Bársony et al. which explored the diagnostic value of continuous, real-time detection of exhaled methane levels as compared to intravital sublingual microscopy, a clinical method already in use; the final goal was to recognize internal hemorrhage in a large animal model. The methane-based detection indicated the presence of bleeding at an early stage and closely followed changes in mesenteric perfusion during hemorrhage and resuscitation as well, with a diagnostic value comparable to the sublingual microcirculatory monitoring. The study therefore presents an interesting consideration for the development of future clinical trials.

A clinical article by Baumbach et al. reports on a novel non-invasive diagnostic device for an *in vivo* assessment of mitochondrial oxygen tension (mitoPO_2_) in humans. The mitochondrial performance parameters were measured in the skin with the protoporphyrin IX-triplet state lifetime technique (PpIX-TSLT), with mitochondrial oxygen consumption changes closely related to gas exchange variables and blood gas parameters during cardiopulmonary exercise testing. As this device allows the direct measurement of oxygen metabolism on the cellular level, it could be a promising tool not only in high-performance sports, but in a clinical setting as well.

## Therapies

Despite fairly extensive studies, the exact mechanistic details behind the side-effects of non-steroid anti-inflammatory drugs (NSAIDs) are not completely understood as yet. To address this, Herminghaus et al. investigated the concentration- and tissue-dependent effects of indomethacin on mitochondrial respiration in different organs, and the data revealed that indomethacin increases the efficacy of oxygen utilization of colonic mitochondria but uncouples hepatic mitochondria, predominantly through complex I. Taken together, the organ-specific, dose-dependent, and complex-specific effects of indomethacin warrant further confirmation with *in vivo* studies.

The next preclinical study focuses on a special subtype of shock. Rewarming victims of hypothermia is often accompanied by reduced cardiac output (CO) and decreased mean arterial blood pressure, which is termed “rewarming shock.” Håheim et al. used a rat model instrumented for measurements of haemodynamic function and organ blood flow (OBF) during stable hypothermia and rewarming to document the effects of two pharmacologic strategies employing vasodilator or inodilator compounds. In terms of blood flow to the brain, both approaches were effective, while CO was elevated to different levels. Mechanistically, the findings indicated that increased vascular resistance is a central element in the complex pathophysiology of rewarming from hypothermia.

The tryptophan-L-kynurenine pathway has been implicated in many inflammatory disorders in the central nervous system, and elevated plasma levels of the glutamate receptor antagonist kynurenic acid (KYNA) have been reported in septic shock patients as well. The next basic science paper by Juhász et al. used a rodent model of intraabdominal sepsis to investigate whether the microcirculation and mitochondrial function is affected by exogenously administered KYNA or a synthetic analog. Both treatment protocols attenuated the deleterious consequences of oxidative/nitrosative stress and resulted in lower inflammatory mediator release. The synthetic KYNA analog SZR-72 markedly improved the key indices of mitochondrial function in liver homogenate after sepsis induction, but only KYNA ameliorated sepsis-related microcirculatory perfusion deficit (a reduction in capillary perfusion and an increase in perfusion heterogeneity), suggesting again a dissociation between the efficacy of mitochondrial and/or microcirculatory treatment strategies in sepsis.

Haemorrhagic shock is the leading cause of preventable death after severe traumas, and melatonin administration during resuscitation improved survival in animal models of haemorrhagic shock and polytrauma. In the next basic science study, Truse et al. tested whether the topical application of melatonin to the gastric mucosa may influence gastric microcirculation in the context of haemorrhagic shock. Melatonin application had no influence on macrohaemodynamic variables, but, besides anti-inflammatory and anti-oxidative actions, the authors demonstrated the significant modulation of the gastric microcirculatory oxygenation as a novel aspect of the tissue-protective effects of melatonin.

The anti-inflammatory potential of ethyl pyruvate (EtP) is associated with reduced systemic inflammation. In relation to trauma, using a clinically-relevant double-hit model of hemorrhage and blunt chest trauma, Dieteren et al. demonstrated a mechanism through which the organ-protective effects of EtP may be linked to the decreased systemic activation of circulating leukocytes. This important line of investigation supports the need for additional studies to further discern the specific dose- and time-dependent influence of EtP on post-traumatic inflammatory response and outcomes.

Traumas and surgeries often present significant challenges to providing accurate resuscitation when blood products or whole blood are not available. To address this anticipated medical need, many possibilities are explored employing solutions that carry oxygen as alternatives. Munoz et al. provided an excellent review of the microvascular pathophysiology of clinical and experimental haemorrhagic shock. The authors innovatively—and convincingly—suggest that the present focus on restoring blood volume and oxygen-carrying capacity should be redirected toward artificial blood substitutes which are designed to restore blood viscosity.

In summary, the studies presented here have provided a significantly greater understanding of the components of shock syndrome that affect critically ill and injured patients. We thank the authors for their original and thought-provoking contributions.

## Author Contributions

All authors listed have made a substantial, direct and intellectual contribution to the work, and approved it for publication.

## Conflict of Interest

The authors declare that the research was conducted in the absence of any commercial or financial relationships that could be construed as a potential conflict of interest.
